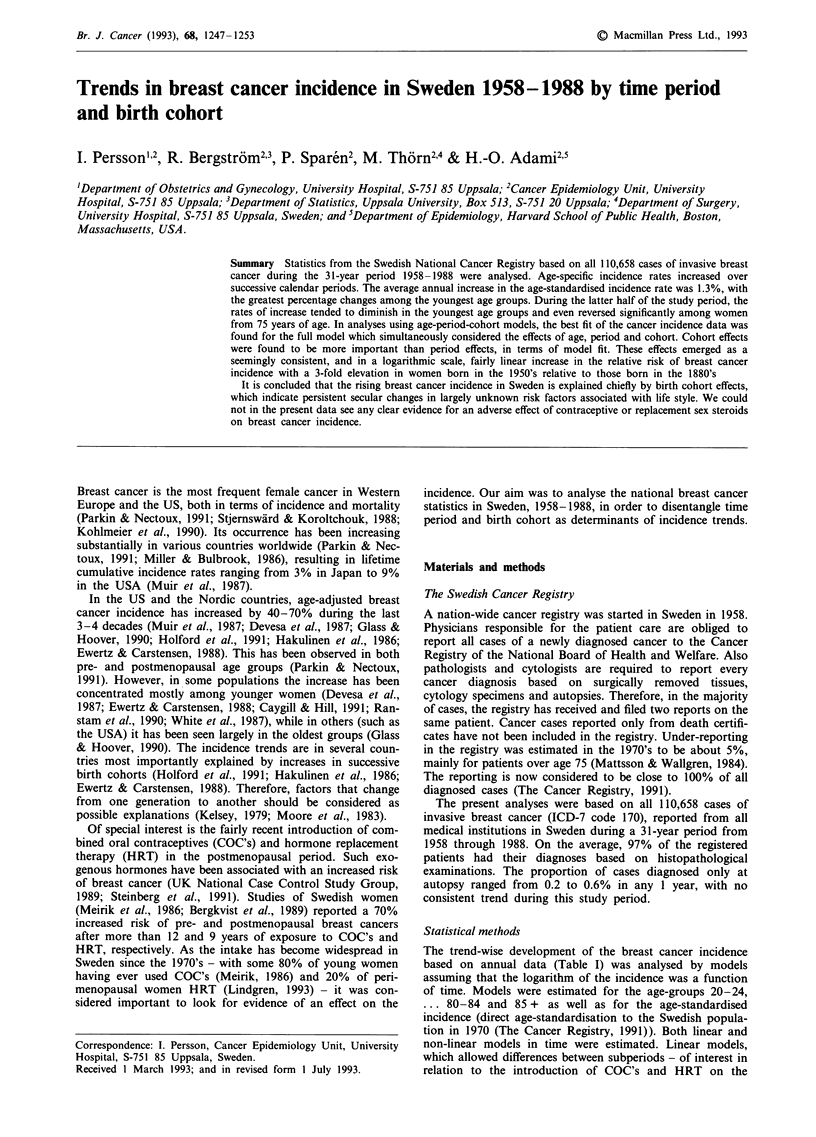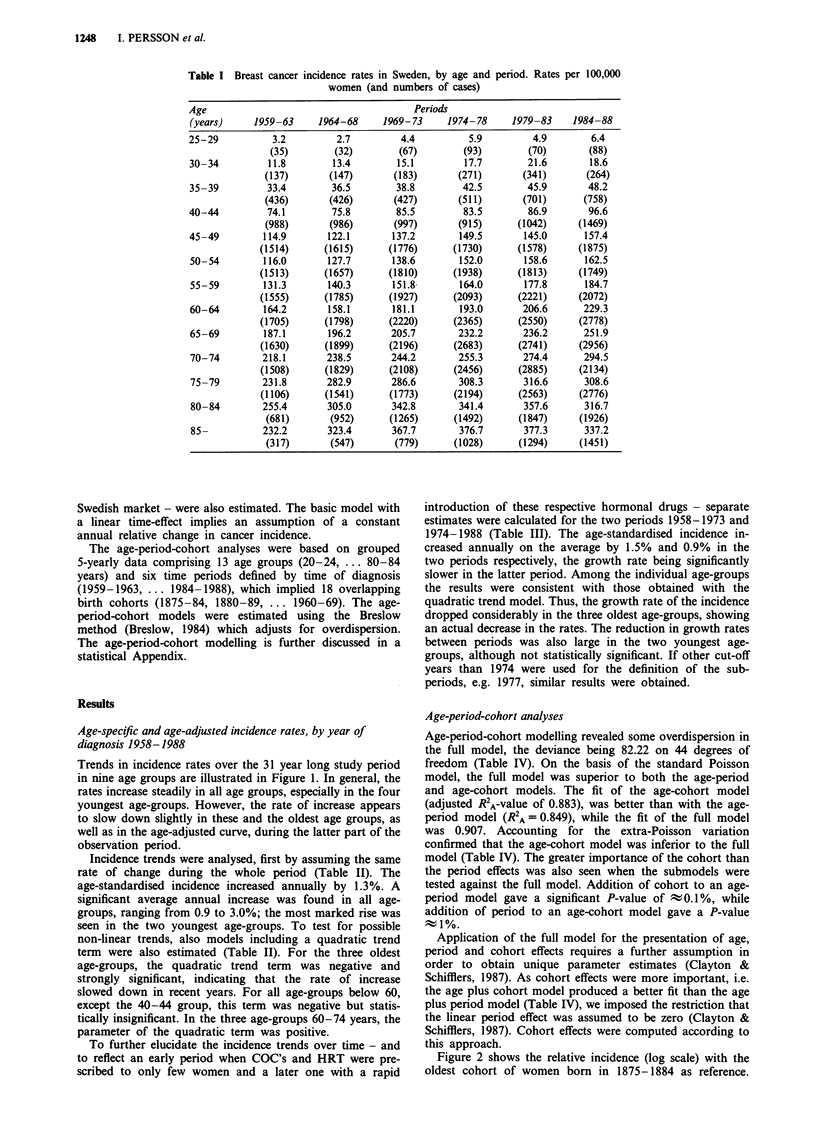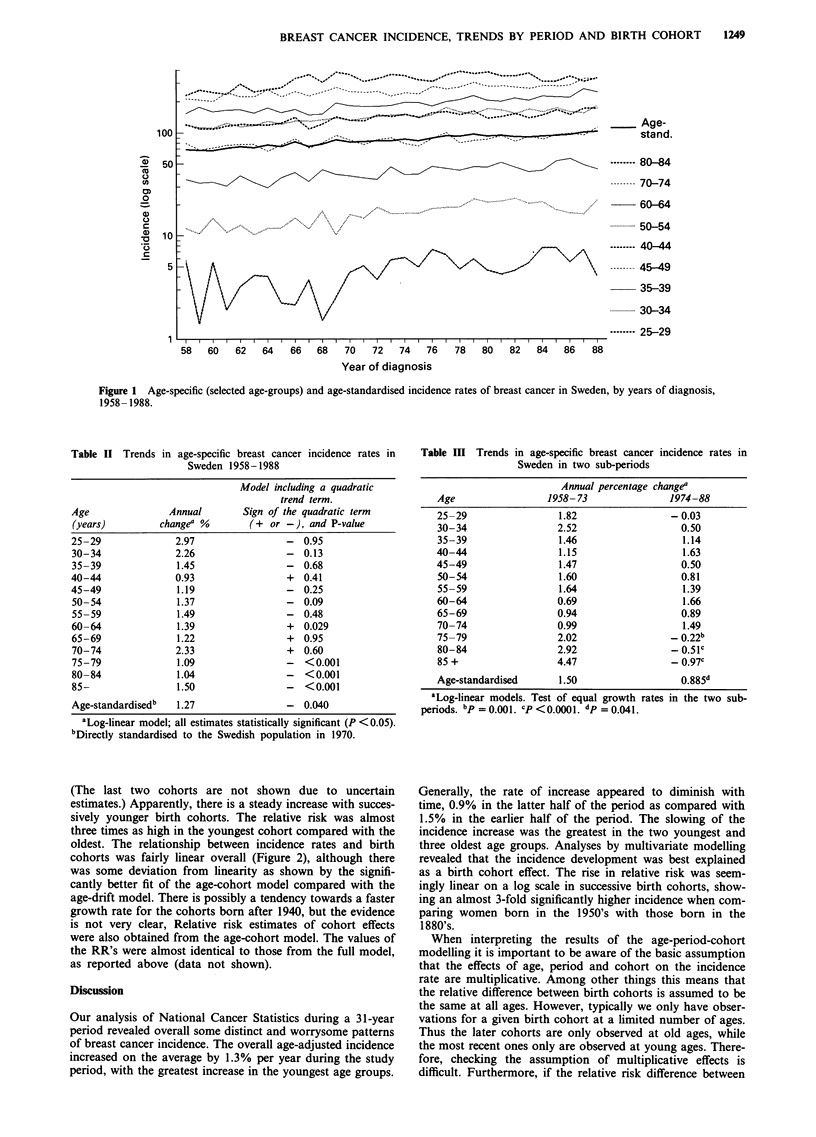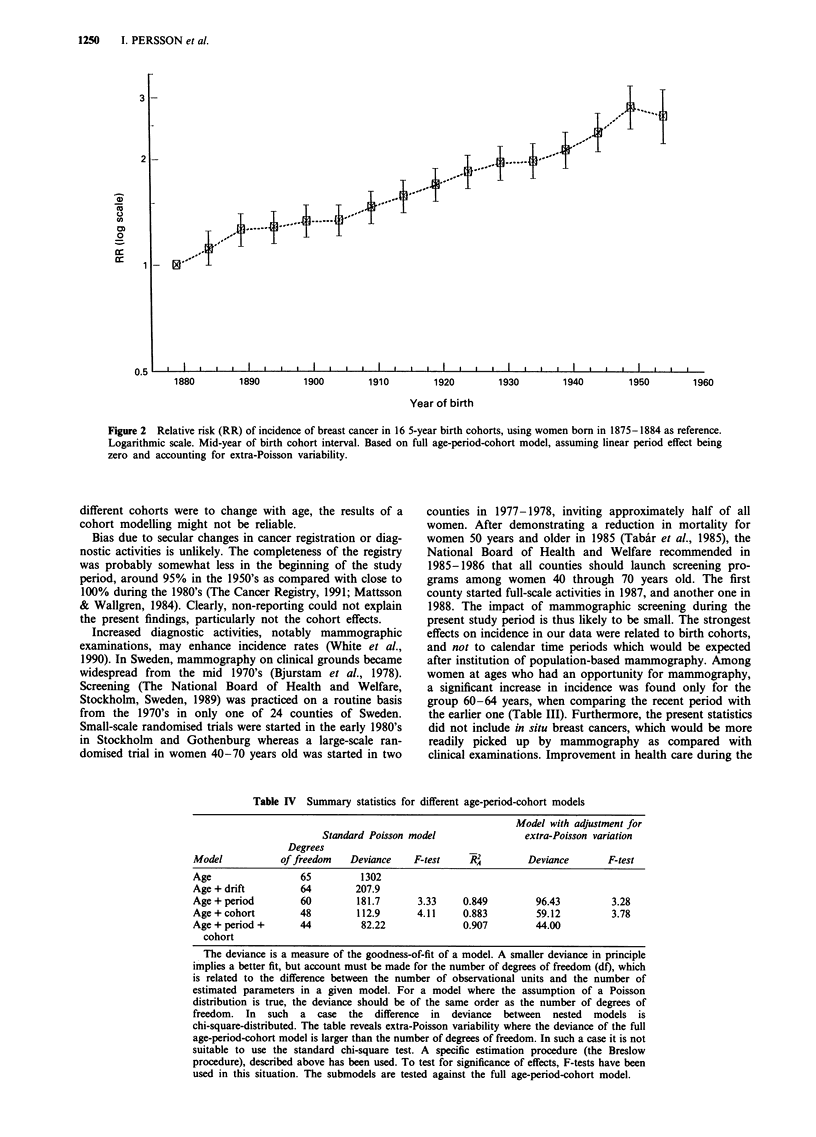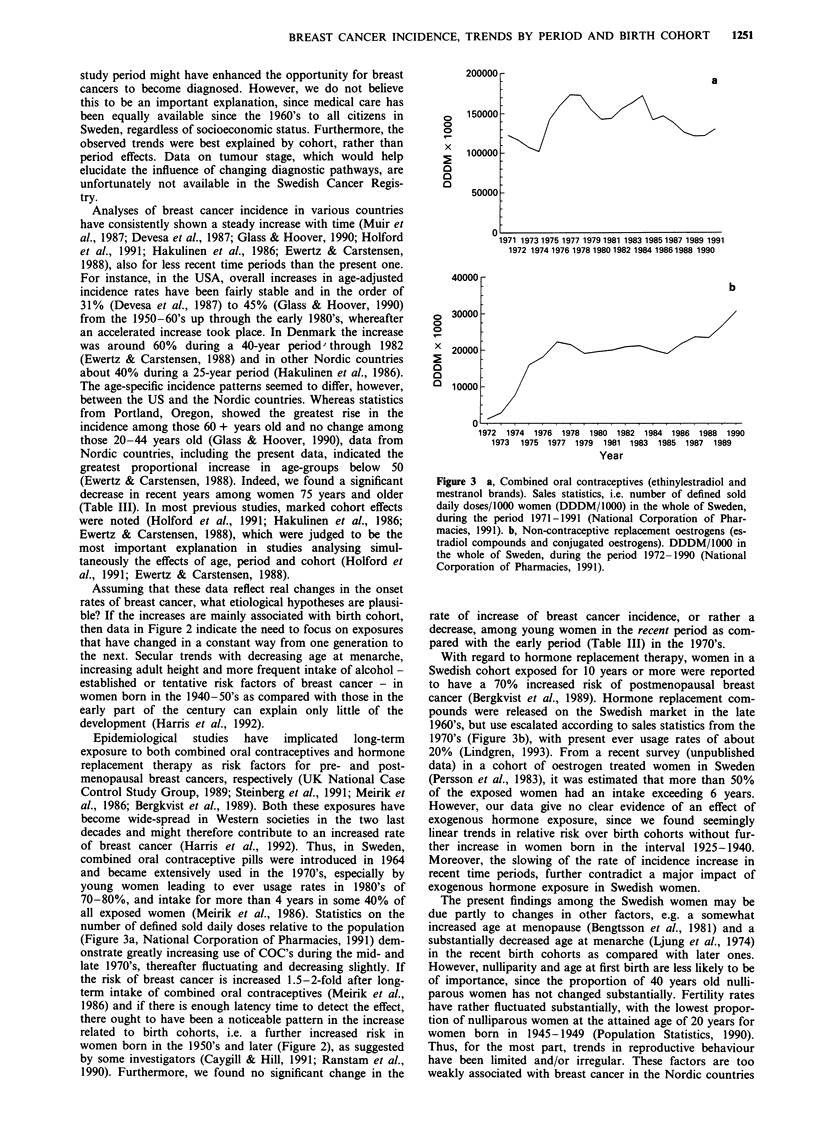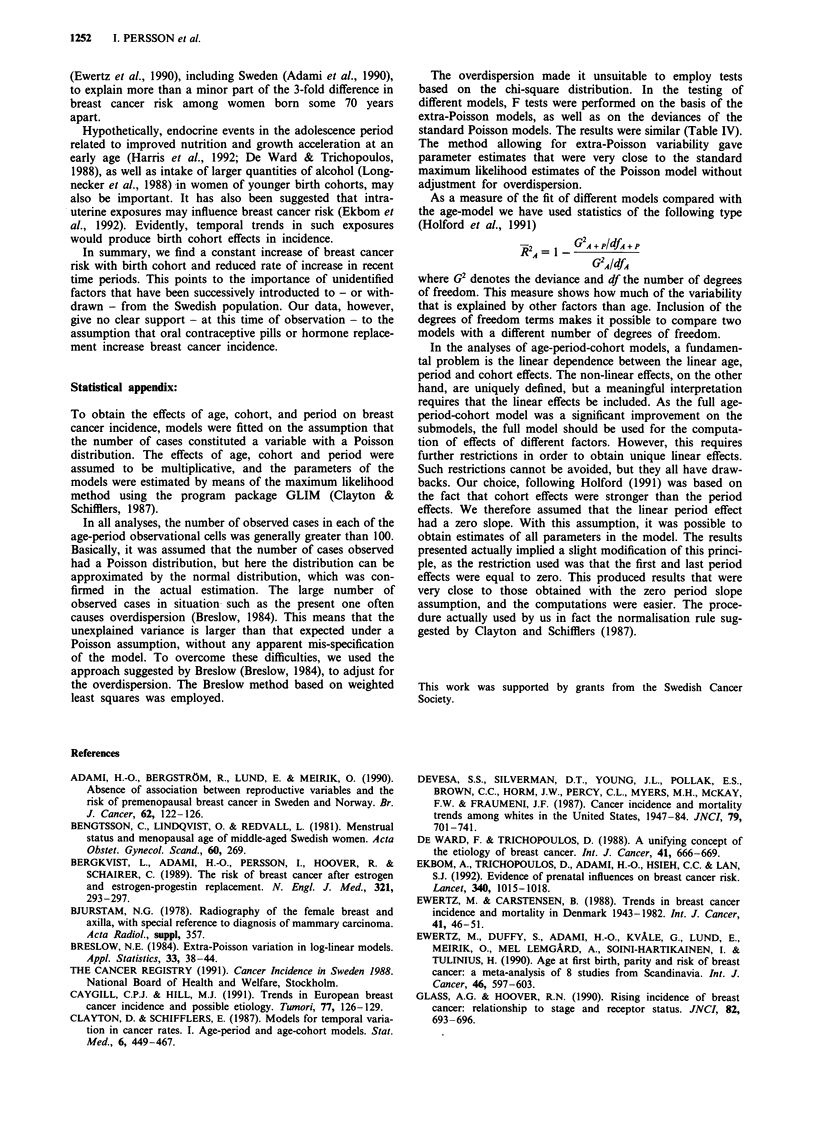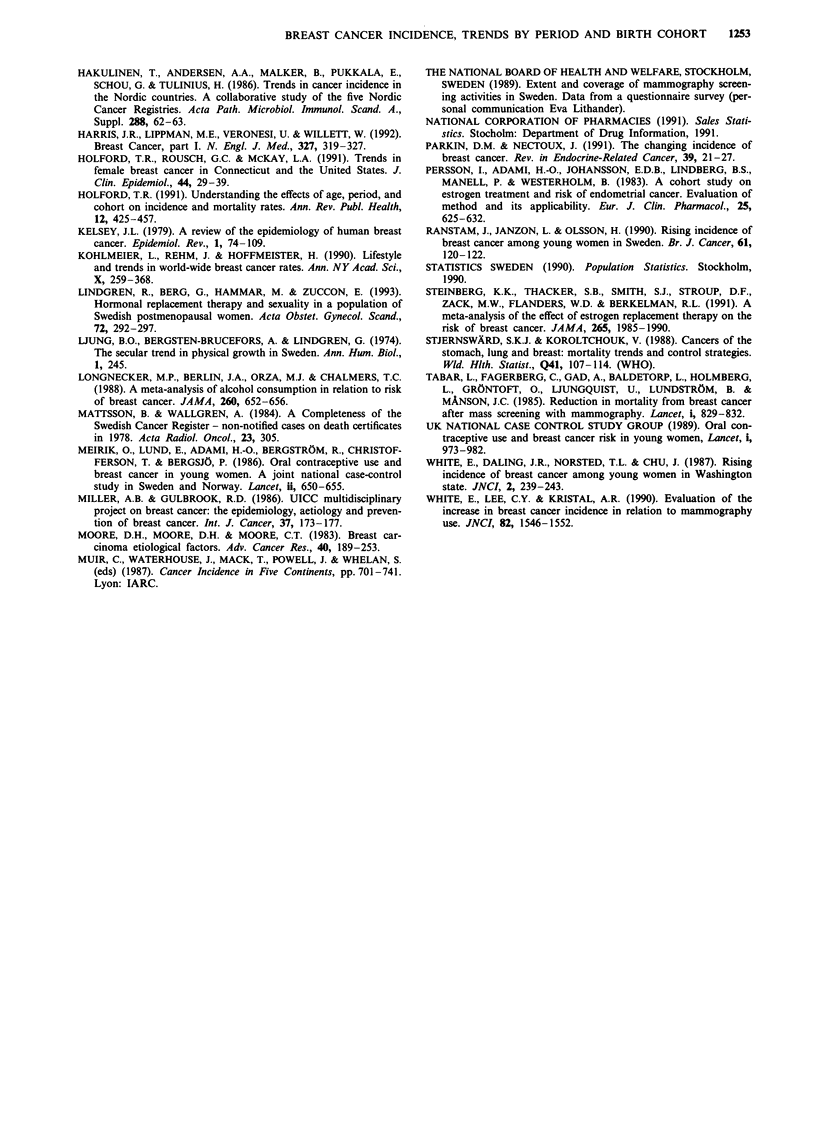# Trends in breast cancer incidence in Sweden 1958-1988 by time period and birth cohort.

**DOI:** 10.1038/bjc.1993.513

**Published:** 1993-12

**Authors:** I. Persson, R. Bergström, P. Sparén, M. Thörn, H. O. Adami

**Affiliations:** Department of Obstetrics and Gynecology, University Hospital, Uppsala, Sweden.

## Abstract

Statistics from the Swedish National Cancer Registry based on all 110,658 cases of invasive breast cancer during the 31-year period 1958-1988 were analysed. Age-specific incidence rates increased over successive calendar periods. The average annual increase in the age-standardised incidence rate was 1.3%, with the greatest percentage changes among the youngest age groups. During the latter half of the study period, the rates of increase tended to diminish in the youngest age groups and even reversed significantly among women from 75 years of age. In analyses using age-period-cohort models, the best fit of the cancer incidence data was found for the full model which simultaneously considered the effects of age, period and cohort. Cohort effects were found to be more important than period effects, in terms of model fit. These effects emerged as a seemingly consistent, and in a logarithmic scale, fairly linear increase in the relative risk of breast cancer incidence with a 3-fold elevation in women born in the 1950's relative to those born in the 1880's. It is concluded that the rising breast cancer incidence in Sweden is explained chiefly by birth cohort effects, which indicate persistent secular changes in largely unknown risk factors associated with life style. We could not in the present data see any clear evidence for an adverse effect of contraceptive or replacement sex steroids on breast cancer incidence.


Br. J. Cancer (1993), 68, 1247–1253 © Macmillan Press Ltd., 1993

### Trends in breast cancer incidence in Sweden 1958–1988 by time period and birth cohort

I. Persson[1,2], R. Bergström[2,3], P. Sparén[2], M. Thörn[2,4] & H.-O. Adami[2,5]

[1]*Department of Obstetrics and Gynecology, University Hospital, S-751 85 Uppsala;* [2]*Cancer Epidemiology Unit, University Hospital, S-751 85 Uppsala;* [3]*Department of Statistics, Uppsala University, Box 513, S-751 20 Uppsala;* [4]*Department of Surgery, University Hospital, S-751 85 Uppsala, Sweden; and* [5]*Department of Epidemiology, Harvard School of Public Health, Boston, Massachusetts, USA.*

**Summary** Statistics from the Swedish National Cancer Registry based on all 110,658 cases of invasive breast cancer during the 31-year period 1958–1988 were analysed. Age-specific incidence rates increased over successive calendar periods. The average annual increase in the age-standardised incidence rate was 1.3%, with the greatest percentage changes among the youngest age groups. During the latter half of the study period, the rates of increase tended to diminish in the youngest age groups and even reversed significantly among women from 75 years of age. In analyses using age-period-cohort models, the best fit of the cancer incidence data was found for the full model which simultaneously considered the effects of age, period and cohort. Cohort effects were found to be more important than period effects, in terms of model fit. These effects emerged as a seemingly consistent, and in a logarithmic scale, fairly linear increase in the relative risk of breast cancer incidence with a 3-fold elevation in women born in the 1950's relative to those born in the 1880's

It is concluded that the rising breast cancer incidence in Sweden is explained chiefly by birth cohort effects, which indicate persistent secular changes in largely unknown risk factors associated with life style. We could not in the present data see any clear evidence for an adverse effect of contraceptive or replacement sex steroids on breast cancer incidence.

Breast cancer is the most frequent female cancer in Western Europe and the US, both in terms of incidence and mortality (Parkin & Nectoux, 1991; Stjernswärd & Koroltchouk, 1988; Kohlmeier *et al.*, 1990). Its occurrence has been increasing substantially in various countries worldwide (Parkin & Nectoux, 1991; Miller & Bulbrook, 1986), resulting in lifetime cumulative incidence rates ranging from 3% in Japan to 9% in the USA (Muir *et al.*, 1987).

In the US and the Nordic countries, age-adjusted breast cancer incidence has increased by 40–70% during the last 3–4 decades (Muir *et al.*, 1987; Devesa *et al.*, 1987; Glass & Hoover, 1990; Holford *et al.*, 1991; Hakulinen *et al.*, 1986; Ewertz & Carstensen, 1988). This has been observed in both pre- and postmenopausal age groups (Parkin & Nectoux, 1991). However, in some populations the increase has been concentrated mostly among younger women (Devesa *et al.*, 1987; Ewertz & Carstensen, 1988; Caygill & Hill, 1991; Ranstam *et al.*, 1990; White *et al.*, 1987), while in others (such as the USA) it has been seen largely in the oldest groups (Glass & Hoover, 1990). The incidence trends are in several countries most importantly explained by increases in successive birth cohorts (Holford *et al.*, 1991; Hakulinen *et al.*, 1986; Ewertz & Carstensen, 1988). Therefore, factors that change from one generation to another should be considered as possible explanations (Kelsey, 1979; Moore *et al.*, 1983).

Of special interest is the fairly recent introduction of combined oral contraceptives (COC's) and hormone replacement therapy (HRT) in the postmenopausal period. Such exogenous hormones have been associated with an increased risk of breast cancer (UK National Case Control Study Group, 1989; Steinberg *et al.*, 1991). Studies of Swedish women (Meirik *et al.*, 1986; Bergkvist *et al.*, 1989) reported a 70% increased risk of pre- and postmenopausal breast cancers after more than 12 and 9 years of exposure to COC's and HRT, respectively. As the intake has become widespread in Sweden since the 1970's – with some 80% of young women having ever used COC's (Meirik, 1986) and 20% of perimenopausal women HRT (Lindgren, 1993) – it was considered important to look for evidence of an effect on the incidence. Our aim was to analyse the national breast cancer statistics in Sweden, 1958–1988, in order to disentangle time period and birth cohort as determinants of incidence trends.

### Materials and methods

### *The Swedish Cancer Registry*

A nation-wide cancer registry was started in Sweden in 1958. Physicians responsible for the patient care are obliged to report all cases of a newly diagnosed cancer to the Cancer Registry of the National Board of Health and Welfare. Also pathologists and cytologists are required to report every cancer diagnosis based on surgically removed tissues, cytology specimens and autopsies. Therefore, in the majority of cases, the registry has received and filed two reports on the same patient. Cancer cases reported only from death certificates have not been included in the registry. Under-reporting in the registry was estimated in the 1970's to be about 5%, mainly for patients over age 75 (Mattsson & Wallgren, 1984). The reporting is now considered to be close to 100% of all diagnosed cases (The Cancer Registry, 1991).

The present analyses were based on all 110,658 cases of invasive breast cancer (ICD-7 code 170), reported from all medical institutions in Sweden during a 31-year period from 1958 through 1988. On the average, 97% of the registered patients had their diagnoses based on histopathological examinations. The proportion of cases diagnosed only at autopsy ranged from 0.2 to 0.6% in any 1 year, with no consistent trend during this study period.

### *Statistical methods*

The trend-wise development of the breast cancer incidence based on annual data (Table I) was analysed by models assuming that the logarithm of the incidence was a function of time. Models were estimated for the age-groups 20–24, ... 80–84 and 85+ as well as for the age-standardised incidence (direct age-standardisation to the Swedish population in 1970 (The Cancer Registry, 1991)). Both linear and non-linear models in time were estimated. Linear models, which allowed differences between subperiods – of interest in relation to the introduction of COC's and HRT on the

Correspondence: I. Persson, Cancer Epidemiology Unit, University Hospital, S-751 85 Uppsala, Sweden.
Received 1 March 1993; and in revised form 1 July 1993.

**Table I** Breast cancer incidence rates in Sweden, by age and period. Rates per 100,000 women (and numbers of cases)

| *Age (years)* | *1959–63* | *1964–68* | *1969–73* | *1974–78* | *1979–83* | *1984–88* |
|---|---|---|---|---|---|---|
| 25–29 | 3.2 | 2.7 | 4.4 | 5.9 | 4.9 | 6.4 |
| | (35) | (32) | (67) | (93) | (70) | (88) |
| 30–34 | 11.8 | 13.4 | 15.1 | 17.7 | 21.6 | 18.6 |
| | (137) | (147) | (183) | (271) | (341) | (264) |
| 35–39 | 33.4 | 36.5 | 38.8 | 42.5 | 45.9 | 48.2 |
| | (436) | (426) | (427) | (511) | (701) | (758) |
| 40–44 | 74.1 | 75.8 | 85.5 | 83.5 | 86.9 | 96.6 |
| | (988) | (986) | (997) | (915) | (1042) | (1469) |
| 45–49 | 114.9 | 122.1 | 137.2 | 149.5 | 145.0 | 157.4 |
| | (1514) | (1615) | (1776) | (1730) | (1578) | (1875) |
| 50–54 | 116.0 | 127.7 | 138.6 | 152.0 | 158.6 | 162.5 |
| | (1513) | (1657) | (1810) | (1938) | (1813) | (1749) |
| 55–59 | 131.3 | 140.3 | 151.8 | 164.0 | 177.8 | 184.7 |
| | (1555) | (1785) | (1927) | (2093) | (2221) | (2072) |
| 60–64 | 164.2 | 158.1 | 181.1 | 193.0 | 206.6 | 229.3 |
| | (1705) | (1798) | (2220) | (2365) | (2550) | (2778) |
| 65–69 | 187.1 | 196.2 | 205.7 | 232.2 | 236.2 | 251.9 |
| | (1630) | (1899) | (2196) | (2683) | (2741) | (2956) |
| 70–74 | 218.1 | 238.5 | 244.2 | 255.3 | 274.4 | 294.5 |
| | (1508) | (1829) | (2108) | (2456) | (2885) | (2134) |
| 75–79 | 231.8 | 282.9 | 286.6 | 308.3 | 316.6 | 308.6 |
| | (1106) | (1541) | (1773) | (2194) | (2563) | (2776) |
| 80–84 | 255.4 | 305.0 | 342.8 | 341.4 | 357.6 | 316.7 |
| | (681) | (952) | (1265) | (1492) | (1847) | (1926) |
| 85– | 232.2 | 323.4 | 367.7 | 376.7 | 377.3 | 337.2 |
| | (317) | (547) | (779) | (1028) | (1294) | (1451) |

Swedish market – were also estimated. The basic model with a linear time-effect implies an assumption of a constant annual relative change in cancer incidence.

The age-period-cohort analyses were based on grouped 5-yearly data comprising 13 age groups (20–24, ... 80–84 years) and six time periods defined by time of diagnosis (1959–1963, ... 1984–1988), which implied 18 overlapping birth cohorts (1875–84, 1880–89, ... 1960–69). The age-period-cohort models were estimated using the Breslow method (Breslow, 1984) which adjusts for overdispersion. The age-period-cohort modelling is further discussed in a statistical Appendix.

### Results

### *Age-specific and age-adjusted incidence rates, by year of diagnosis 1958–1988*

Trends in incidence rates over the 31 year long study period in nine age groups are illustrated in Figure 1. In general, the rates increase steadily in all age groups, especially in the four youngest age-groups. However, the rate of increase appears to slow down slightly in these and the oldest age groups, as well as in the age-adjusted curve, during the latter part of the observation period.

Incidence trends were analysed, first by assuming the same rate of change during the whole period (Table II). The age-standardised incidence increased annually by 1.3%. A significant average annual increase was found in all age-groups, ranging from 0.9 to 3.0%; the most marked rise was seen in the two youngest age-groups. To test for possible non-linear trends, also models including a quadratic trend term were also estimated (Table II). For the three oldest age-groups, the quadratic trend term was negative and strongly significant, indicating that the rate of increase slowed down in recent years. For all age-groups below 60, except the 40–44 group, this term was negative but statistically insignificant. In the three age-groups 60–74 years, the parameter of the quadratic term was positive.

To further elucidate the incidence trends over time – and to reflect an early period when COC's and HRT were prescribed to only few women and a later one with a rapid introduction of these respective hormonal drugs – separate estimates were calculated for the two periods 1958–1973 and 1974–1988 (Table III). The age-standardised incidence increased annually on the average by 1.5% and 0.9% in the two periods respectively, the growth rate being significantly slower in the latter period. Among the individual age-groups the results were consistent with those obtained with the quadratic trend model. Thus, the growth rate of the incidence dropped considerably in the three oldest age-groups, showing an actual decrease in the rates. The reduction in growth rates between periods was also large in the two youngest age-groups, although not statistically significant. If other cut-off years than 1974 were used for the definition of the sub-periods, e.g. 1977, similar results were obtained.

### *Age-period-cohort analyses*

Age-period-cohort modelling revealed some overdispersion in the full model, the deviance being 82.22 on 44 degrees of freedom (Table IV). On the basis of the standard Poisson model, the full model was superior to both the age-period and age-cohort models. The fit of the age-cohort model (adjusted $R^2_A$-value of 0.883), was better than with the age-period model ($R^2_A = 0.849$), while the fit of the full model was 0.907. Accounting for the extra-Poisson variation confirmed that the age-cohort model was inferior to the full model (Table IV). The greater importance of the cohort than the period effects was also seen when the submodels were tested against the full model. Addition of cohort to an age-period model gave a significant $P$-value of $\approx 0.1\%$, while addition of period to an age-cohort model gave a $P$-value $\approx 1\%$.

Application of the full model for the presentation of age, period and cohort effects requires a further assumption in order to obtain unique parameter estimates (Clayton & Schifflers, 1987). As cohort effects were more important, i.e. the age plus cohort model produced a better fit than the age plus period model (Table IV), we imposed the restriction that the linear period effect was assumed to be zero (Clayton & Schifflers, 1987). Cohort effects were computed according to this approach.

Figure 2 shows the relative incidence (log scale) with the oldest cohort of women born in 1875–1884 as reference.

**Figure 1** Age-specific (selected age-groups) and age-standardised incidence rates of breast cancer in Sweden, by years of diagnosis, 1958–1988.

**Table II** Trends in age-specific breast cancer incidence rates in Sweden 1958–1988

| *Age (years)* | *Annual change[a] %* | *Model including a quadratic trend term. Sign of the quadratic term (+ or −), and P-value* |
|---|---|---|
| 25–29 | 2.97 | − 0.95 |
| 30–34 | 2.26 | − 0.13 |
| 35–39 | 1.45 | − 0.68 |
| 40–44 | 0.93 | + 0.41 |
| 45–49 | 1.19 | − 0.25 |
| 50–54 | 1.37 | − 0.09 |
| 55–59 | 1.49 | − 0.48 |
| 60–64 | 1.39 | + 0.029 |
| 65–69 | 1.22 | + 0.95 |
| 70–74 | 2.33 | + 0.60 |
| 75–79 | 1.09 | − <0.001 |
| 80–84 | 1.04 | − <0.001 |
| 85– | 1.50 | − <0.001 |
| Age-standardised[b] | 1.27 | − 0.040 |

[a]Log-linear model; all estimates statistically significant ($P < 0.05$). [b]Directly standardised to the Swedish population in 1970.

**Table III** Trends in age-specific breast cancer incidence rates in Sweden in two sub-periods

| *Age* | *Annual percentage change[a]* 1958–73 | 1974–88 |
|---|---|---|
| 25–29 | 1.82 | − 0.03 |
| 30–34 | 2.52 | 0.50 |
| 35–39 | 1.46 | 1.14 |
| 40–44 | 1.15 | 1.63 |
| 45–49 | 1.47 | 0.50 |
| 50–54 | 1.60 | 0.81 |
| 55–59 | 1.64 | 1.39 |
| 60–64 | 0.69 | 1.66 |
| 65–69 | 0.94 | 0.89 |
| 70–74 | 0.99 | 1.49 |
| 75–79 | 2.02 | − 0.22[b] |
| 80–84 | 2.92 | − 0.51[c] |
| 85 + | 4.47 | − 0.97[c] |
| Age-standardised | 1.50 | 0.885[d] |

[a]Log-linear models. Test of equal growth rates in the two sub-periods. [b]$P = 0.001$. [c]$P < 0.0001$. [d]$P = 0.041$.

(The last two cohorts are not shown due to uncertain estimates.) Apparently, there is a steady increase with successively younger birth cohorts. The relative risk was almost three times as high in the youngest cohort compared with the oldest. The relationship between incidence rates and birth cohorts was fairly linear overall (Figure 2), although there was some deviation from linearity as shown by the significantly better fit of the age-cohort model compared with the age-drift model. There is possibly a tendency towards a faster growth rate for the cohorts born after 1940, but the evidence is not very clear, Relative risk estimates of cohort effects were also obtained from the age-cohort model. The values of the RR's were almost identical to those from the full model, as reported above (data not shown).

### Discussion

Our analysis of National Cancer Statistics during a 31-year period revealed overall some distinct and worrysome patterns of breast cancer incidence. The overall age-adjusted incidence increased on the average by 1.3% per year during the study period, with the greatest increase in the youngest age groups. Generally, the rate of increase appeared to diminish with time, 0.9% in the latter half of the period as compared with 1.5% in the earlier half of the period. The slowing of the incidence increase was the greatest in the two youngest and three oldest age groups. Analyses by multivariate modelling revealed that the incidence development was best explained as a birth cohort effect. The rise in relative risk was seemingly linear on a log scale in successive birth cohorts, showing an almost 3-fold significantly higher incidence when comparing women born in the 1950's with those born in the 1880's.

When interpreting the results of the age-period-cohort modelling it is important to be aware of the basic assumption that the effects of age, period and cohort on the incidence rate are multiplicative. Among other things this means that the relative difference between birth cohorts is assumed to be the same at all ages. However, typically we only have observations for a given birth cohort at a limited number of ages. Thus the later cohorts are only observed at old ages, while the most recent ones only are observed at young ages. Therefore, checking the assumption of multiplicative effects is difficult. Furthermore, if the relative risk difference between

**Figure 2** Relative risk (RR) of incidence of breast cancer in 16 5-year birth cohorts, using women born in 1875–1884 as reference. Logarithmic scale. Mid-year of birth cohort interval. Based on full age-period-cohort model, assuming linear period effect being zero and accounting for extra-Poisson variability.

different cohorts were to change with age, the results of a cohort modelling might not be reliable.

Bias due to secular changes in cancer registration or diagnostic activities is unlikely. The completeness of the registry was probably somewhat less in the beginning of the study period, around 95% in the 1950's as compared with close to 100% during the 1980's (The Cancer Registry, 1991; Mattsson & Wallgren, 1984). Clearly, non-reporting could not explain the present findings, particularly not the cohort effects.

Increased diagnostic activities, notably mammographic examinations, may enhance incidence rates (White *et al.*, 1990). In Sweden, mammography on clinical grounds became widespread from the mid 1970's (Bjurstam *et al.*, 1978). Screening (The National Board of Health and Welfare, Stockholm, Sweden, 1989) was practiced on a routine basis from the 1970's in only one of 24 counties of Sweden. Small-scale randomised trials were started in the early 1980's in Stockholm and Gothenburg whereas a large-scale randomised trial in women 40–70 years old was started in two counties in 1977–1978, inviting approximately half of all women. After demonstrating a reduction in mortality for women 50 years and older in 1985 (Tabár *et al.*, 1985), the National Board of Health and Welfare recommended in 1985–1986 that all counties should launch screening programs among women 40 through 70 years old. The first county started full-scale activities in 1987, and another one in 1988. The impact of mammographic screening during the present study period is thus likely to be small. The strongest effects on incidence in our data were related to birth cohorts, and *not* to calendar time periods which would be expected after institution of population-based mammography. Among women at ages who had an opportunity for mammography, a significant increase in incidence was found only for the group 60–64 years, when comparing the recent period with the earlier one (Table III). Furthermore, the present statistics did not include *in situ* breast cancers, which would be more readily picked up by mammography as compared with clinical examinations. Improvement in health care during the

**Table IV** Summary statistics for different age-period-cohort models

| | *Standard Poisson model* | | | | *Model with adjustment for extra-Poisson variation* | |
|---|---|---|---|---|---|---|
| *Model* | *Degrees of freedom* | *Deviance* | *F-test* | $\bar{R}^2_A$ | *Deviance* | *F-test* |
| Age | 65 | 1302 | | | | |
| Age + drift | 64 | 207.9 | | | | |
| Age + period | 60 | 181.7 | 3.33 | 0.849 | 96.43 | 3.28 |
| Age + cohort | 48 | 112.9 | 4.11 | 0.883 | 59.12 | 3.78 |
| Age + period + cohort | 44 | 82.22 | | 0.907 | 44.00 | |

The deviance is a measure of the goodness-of-fit of a model. A smaller deviance in principle implies a better fit, but account must be made for the number of degrees of freedom (df), which is related to the difference between the number of observational units and the number of estimated parameters in a given model. For a model where the assumption of a Poisson distribution is true, the deviance should be of the same order as the number of degrees of freedom. In such a case the difference in deviance between nested models is chi-square-distributed. The table reveals extra-Poisson variability where the deviance of the full age-period-cohort model is larger than the number of degrees of freedom. In such a case it is not suitable to use the standard chi-square test. A specific estimation procedure (the Breslow procedure), described above has been used. To test for significance of effects, F-tests have been used in this situation. The submodels are tested against the full age-period-cohort model.

study period might have enhanced the opportunity for breast cancers to become diagnosed. However, we do not believe this to be an important explanation, since medical care has been equally available since the 1960's to all citizens in Sweden, regardless of socioeconomic status. Furthermore, the observed trends were best explained by cohort, rather than period effects. Data on tumour stage, which would help elucidate the influence of changing diagnostic pathways, are unfortunately not available in the Swedish Cancer Registry.

Analyses of breast cancer incidence in various countries have consistently shown a steady increase with time (Muir *et al.*, 1987; Devesa *et al.*, 1987; Glass & Hoover, 1990; Holford *et al.*, 1991; Hakulinen *et al.*, 1986; Ewertz & Carstensen, 1988), also for less recent time periods than the present one. For instance, in the USA, overall increases in age-adjusted incidence rates have been fairly stable and in the order of 31% (Devesa *et al.*, 1987) to 45% (Glass & Hoover, 1990) from the 1950-60's up through the early 1980's, whereafter an accelerated increase took place. In Denmark the increase was around 60% during a 40-year period' through 1982 (Ewertz & Carstensen, 1988) and in other Nordic countries about 40% during a 25-year period (Hakulinen *et al.*, 1986). The age-specific incidence patterns seemed to differ, however, between the US and the Nordic countries. Whereas statistics from Portland, Oregon, showed the greatest rise in the incidence among those 60 + years old and no change among those 20-44 years old (Glass & Hoover, 1990), data from Nordic countries, including the present data, indicated the greatest proportional increase in age-groups below 50 (Ewertz & Carstensen, 1988). Indeed, we found a significant decrease in recent years among women 75 years and older (Table III). In most previous studies, marked cohort effects were noted (Holford *et al.*, 1991; Hakulinen *et al.*, 1986; Ewertz & Carstensen, 1988), which were judged to be the most important explanation in studies analysing simultaneously the effects of age, period and cohort (Holford *et al.*, 1991; Ewertz & Carstensen, 1988).

Assuming that these data reflect real changes in the onset rates of breast cancer, what etiological hypotheses are plausible? If the increases are mainly associated with birth cohort, then data in Figure 2 indicate the need to focus on exposures that have changed in a constant way from one generation to the next. Secular trends with decreasing age at menarche, increasing adult height and more frequent intake of alcohol - established or tentative risk factors of breast cancer - in women born in the 1940-50's as compared with those in the early part of the century can explain only little of the development (Harris *et al.*, 1992).

Epidemiological studies have implicated long-term exposure to both combined oral contraceptives and hormone replacement therapy as risk factors for pre- and postmenopausal breast cancers, respectively (UK National Case Control Study Group, 1989; Steinberg *et al.*, 1991; Meirik *et al.*, 1986; Bergkvist *et al.*, 1989). Both these exposures have become wide-spread in Western societies in the two last decades and might therefore contribute to an increased rate of breast cancer (Harris *et al.*, 1992). Thus, in Sweden, combined oral contraceptive pills were introduced in 1964 and became extensively used in the 1970's, especially by young women leading to ever usage rates in 1980's of 70-80%, and intake for more than 4 years in some 40% of all exposed women (Meirik *et al.*, 1986). Statistics on the number of defined sold daily doses relative to the population (Figure 3a, National Corporation of Pharmacies, 1991) demonstrate greatly increasing use of COC's during the mid- and late 1970's, thereafter fluctuating and decreasing slightly. If the risk of breast cancer is increased 1.5-2-fold after long-term intake of combined oral contraceptives (Meirik *et al.*, 1986) and if there is enough latency time to detect the effect, there ought to have been a noticeable pattern in the increase related to birth cohorts, i.e. a further increased risk in women born in the 1950's and later (Figure 2), as suggested by some investigators (Caygill & Hill, 1991; Ranstam *et al.*, 1990). Furthermore, we found no significant change in the

**Figure 3** **a**, Combined oral contraceptives (ethinylestradiol and mestranol brands). Sales statistics, i.e. number of defined sold daily doses/1000 women (DDDM/1000) in the whole of Sweden, during the period 1971-1991 (National Corporation of Pharmacies, 1991). **b**, Non-contraceptive replacement oestrogens (estradiol compounds and conjugated oestrogens). DDDM/1000 in the whole of Sweden, during the period 1972-1990 (National Corporation of Pharmacies, 1991).

rate of increase of breast cancer incidence, or rather a decrease, among young women in the *recent* period as compared with the early period (Table III) in the 1970's.

With regard to hormone replacement therapy, women in a Swedish cohort exposed for 10 years or more were reported to have a 70% increased risk of postmenopausal breast cancer (Bergkvist *et al.*, 1989). Hormone replacement compounds were released on the Swedish market in the late 1960's, but use escalated according to sales statistics from the 1970's (Figure 3b), with present ever usage rates of about 20% (Lindgren, 1993). From a recent survey (unpublished data) in a cohort of oestrogen treated women in Sweden (Persson *et al.*, 1983), it was estimated that more than 50% of the exposed women had an intake exceeding 6 years. However, our data give no clear evidence of an effect of exogenous hormone exposure, since we found seemingly linear trends in relative risk over birth cohorts without further increase in women born in the interval 1925-1940. Moreover, the slowing of the rate of incidence increase in recent time periods, further contradict a major impact of exogenous hormone exposure in Swedish women.

The present findings among the Swedish women may be due partly to changes in other factors, e.g. a somewhat increased age at menopause (Bengtsson *et al.*, 1981) and a substantially decreased age at menarche (Ljung *et al.*, 1974) in the recent birth cohorts as compared with later ones. However, nulliparity and age at first birth are less likely to be of importance, since the proportion of 40 years old nulliparous women has not changed substantially. Fertility rates have rather fluctuated substantially, with the lowest proportion of nulliparous women at the attained age of 20 years for women born in 1945-1949 (Population Statistics, 1990). Thus, for the most part, trends in reproductive behaviour have been limited and/or irregular. These factors are too weakly associated with breast cancer in the Nordic countries

(Ewertz et al., 1990), including Sweden (Adami et al., 1990), to explain more than a minor part of the 3-fold difference in breast cancer risk among women born some 70 years apart.

Hypothetically, endocrine events in the adolescence period related to improved nutrition and growth acceleration at an early age (Harris et al., 1992; De Ward & Trichopoulos, 1988), as well as intake of larger quantities of alcohol (Longnecker et al., 1988) in women of younger birth cohorts, may also be important. It has also been suggested that intrauterine exposures may influence breast cancer risk (Ekbom et al., 1992). Evidently, temporal trends in such exposures would produce birth cohort effects in incidence.

In summary, we find a constant increase of breast cancer risk with birth cohort and reduced rate of increase in recent time periods. This points to the importance of unidentified factors that have been successively introducted to – or withdrawn – from the Swedish population. Our data, however, give no clear support – at this time of observation – to the assumption that oral contraceptive pills or hormone replacement increase breast cancer incidence.

### Statistical appendix:

To obtain the effects of age, cohort, and period on breast cancer incidence, models were fitted on the assumption that the number of cases constituted a variable with a Poisson distribution. The effects of age, cohort and period were assumed to be multiplicative, and the parameters of the models were estimated by means of the maximum likelihood method using the program package GLIM (Clayton & Schifflers, 1987).

In all analyses, the number of observed cases in each of the age-period observational cells was generally greater than 100. Basically, it was assumed that the number of cases observed had a Poisson distribution, but here the distribution can be approximated by the normal distribution, which was confirmed in the actual estimation. The large number of observed cases in situation such as the present one often causes overdispersion (Breslow, 1984). This means that the unexplained variance is larger than that expected under a Poisson assumption, without any apparent mis-specification of the model. To overcome these difficulties, we used the approach suggested by Breslow (Breslow, 1984), to adjust for the overdispersion. The Breslow method based on weighted least squares was employed.

The overdispersion made it unsuitable to employ tests based on the chi-square distribution. In the testing of different models, F tests were performed on the basis of the extra-Poisson models, as well as on the deviances of the standard Poisson models. The results were similar (Table IV). The method allowing for extra-Poisson variability gave parameter estimates that were very close to the standard maximum likelihood estimates of the Poisson model without adjustment for overdispersion.

As a measure of the fit of different models compared with the age-model we have used statistics of the following type (Holford et al., 1991)

$$\bar{R}^2_A = 1 - \frac{G^2_{A+P}/df_{A+P}}{G^2_A/df_A}$$

where $G^2$ denotes the deviance and $df$ the number of degrees of freedom. This measure shows how much of the variability that is explained by other factors than age. Inclusion of the degrees of freedom terms makes it possible to compare two models with a different number of degrees of freedom.

In the analyses of age-period-cohort models, a fundamental problem is the linear dependence between the linear age, period and cohort effects. The non-linear effects, on the other hand, are uniquely defined, but a meaningful interpretation requires that the linear effects be included. As the full age-period-cohort model was a significant improvement on the submodels, the full model should be used for the computation of effects of different factors. However, this requires further restrictions in order to obtain unique linear effects. Such restrictions cannot be avoided, but they all have drawbacks. Our choice, following Holford (1991) was based on the fact that cohort effects were stronger than the period effects. We therefore assumed that the linear period effect had a zero slope. With this assumption, it was possible to obtain estimates of all parameters in the model. The results presented actually implied a slight modification of this principle, as the restriction used was that the first and last period effects were equal to zero. This produced results that were very close to those obtained with the zero period slope assumption, and the computations were easier. The procedure actually used by us in fact the normalisation rule suggested by Clayton and Schifflers (1987).

This work was supported by grants from the Swedish Cancer Society.

### References

ADAMI, H.-O., BERGSTRÖM, R., LUND, E. & MEIRIK, O. (1990). Absence of association between reproductive variables and the risk of premenopausal breast cancer in Sweden and Norway. *Br. J. Cancer*, **62**, 122–126.

BENGTSSON, C., LINDQVIST, O. & REDVALL, L. (1981). Menstrual status and menopausal age of middle-aged Swedish women. *Acta Obstet. Gynecol. Scand.*, **60**, 269.

BERGKVIST, L., ADAMI, H.-O., PERSSON, I., HOOVER, R. & SCHAIRER, C. (1989). The risk of breast cancer after estrogen and estrogen-progestin replacement. *N. Engl. J. Med.*, **321**, 293–297.

BJURSTAM, N.G. (1978). Radiography of the female breast and axilla, with special reference to diagnosis of mammary carcinoma. *Acta Radiol.*, **suppl**, 357.

BRESLOW, N.E. (1984). Extra-Poisson variation in log-linear models. *Appl. Statistics*, **33**, 38–44.

THE CANCER REGISTRY (1991). *Cancer Incidence in Sweden 1988*. National Board of Health and Welfare, Stockholm.

CAYGILL, C.P.J. & HILL, M.J. (1991). Trends in European breast cancer incidence and possible etiology. *Tumori*, **77**, 126–129.

CLAYTON, D. & SCHIFFLERS, E. (1987). Models for temporal variation in cancer rates. I. Age-period and age-cohort models. *Stat. Med.*, **6**, 449–467.

DEVESA, S.S., SILVERMAN, D.T., YOUNG, J.L., POLLAK, E.S., BROWN, C.C., HORM, J.W., PERCY, C.L., MYERS, M.H., MCKAY, F.W. & FRAUMENI, J.F. (1987). Cancer incidence and mortality trends among whites in the United States, 1947–84. *JNCI*, **79**, 701–741.

DE WARD, F. & TRICHOPOULOS, D. (1988). A unifying concept of the etiology of breast cancer. *Int. J. Cancer*, **41**, 666–669.

EKBOM, A., TRICHOPOULOS, D., ADAMI, H.-O., HSIEH, C.C. & LAN, S.J. (1992). Evidence of prenatal influences on breast cancer risk. *Lancet*, **340**, 1015–1018.

EWERTZ, M. & CARSTENSEN, B. (1988). Trends in breast cancer incidence and mortality in Denmark 1943–1982. *Int. J. Cancer*, **41**, 46–51.

EWERTZ, M., DUFFY, S., ADAMI, H.-O., KVÅLE, G., LUND, E., MEIRIK, O., MEL LEMGÅRD, A., SOINI-HARTIKAINEN, I. & TULINIUS, H. (1990). Age at first birth, parity and risk of breast cancer: a meta-analysis of 8 studies from Scandinavia. *Int. J. Cancer*, **46**, 597–603.

GLASS, A.G. & HOOVER, R.N. (1990). Rising incidence of breast cancer: relationship to stage and receptor status. *JNCI*, **82**, 693–696.

HAKULINEN, T., ANDERSEN, A.A., MALKER, B., PUKKALA, E., SCHOU, G. & TULINIUS, H. (1986). Trends in cancer incidence in the Nordic countries. A collaborative study of the five Nordic Cancer Registries. *Acta Path. Microbiol. Immunol. Scand. A.*, Suppl. **288**, 62–63.

HARRIS, J.R., LIPPMAN, M.E., VERONESI, U. & WILLETT, W. (1992). Breast Cancer, part I. *N. Engl. J. Med.*, **327**, 319–327.

HOLFORD, T.R., ROUSCH, G.C. & MCKAY, L.A. (1991). Trends in female breast cancer in Connecticut and the United States. *J. Clin. Epidemiol.*, **44**, 29–39.

HOLFORD, T.R. (1991). Understanding the effects of age, period, and cohort on incidence and mortality rates. *Ann. Rev. Publ. Health*, **12**, 425–457.

KELSEY, J.L. (1979). A review of the epidemiology of human breast cancer. *Epidemiol. Rev.*, **1**, 74–109.

KOHLMEIER, L., REHM, J. & HOFFMEISTER, H. (1990). Lifestyle and trends in world-wide breast cancer rates. *Ann. NY Acad. Sci.*, **X**, 259–368.

LINDGREN, R., BERG, G., HAMMAR, M. & ZUCCON, E. (1993). Hormonal replacement therapy and sexuality in a population of Swedish postmenopausal women. *Acta Obstet. Gynecol. Scand.*, **72**, 292–297.

LJUNG, B.O., BERGSTEN-BRUCEFORS, A. & LINDGREN, G. (1974). The secular trend in physical growth in Sweden. *Ann. Hum. Biol.*, **1**, 245.

LONGNECKER, M.P., BERLIN, J.A., ORZA, M.J. & CHALMERS, T.C. (1988). A meta-analysis of alcohol consumption in relation to risk of breast cancer. *JAMA*, **260**, 652–656.

MATTSSON, B. & WALLGREN, A. (1984). A Completeness of the Swedish Cancer Register – non-notified cases on death certificates in 1978. *Acta Radiol. Oncol.*, **23**, 305.

MEIRIK, O., LUND, E., ADAMI, H.-O., BERGSTRÖM, R., CHRISTOFFERSON, T. & BERGSJÖ, P. (1986). Oral contraceptive use and breast cancer in young women. A joint national case-control study in Sweden and Norway. *Lancet*, **ii**, 650–655.

MILLER, A.B. & GULBROOK, R.D. (1986). UICC multidisciplinary project on breast cancer: the epidemiology, aetiology and prevention of breast cancer. *Int. J. Cancer*, **37**, 173–177.

MOORE, D.H., MOORE, D.H. & MOORE, C.T. (1983). Breast carcinoma etiological factors. *Adv. Cancer Res.*, **40**, 189–253.

MUIR, C., WATERHOUSE, J., MACK, T., POWELL, J. & WHELAN, S. (eds) (1987). *Cancer Incidence in Five Continents*, pp. 701–741. Lyon: IARC.

THE NATIONAL BOARD OF HEALTH AND WELFARE, STOCKHOLM, SWEDEN (1989). Extent and coverage of mammography screening activities in Sweden. Data from a questionnaire survey (personal communication Eva Lithander).

NATIONAL CORPORATION OF PHARMACIES (1991). *Sales Statistics*. Stocholm: Department of Drug Information, 1991.

PARKIN, D.M. & NECTOUX, J. (1991). The changing incidence of breast cancer. *Rev. in Endocrine-Related Cancer*, **39**, 21–27.

PERSSON, I., ADAMI, H.-O., JOHANSSON, E.D.B., LINDBERG, B.S., MANELL, P. & WESTERHOLM, B. (1983). A cohort study on estrogen treatment and risk of endometrial cancer. Evaluation of method and its applicability. *Eur. J. Clin. Pharmacol.*, **25**, 625–632.

RANSTAM, J., JANZON, L. & OLSSON, H. (1990). Rising incidence of breast cancer among young women in Sweden. *Br. J. Cancer*, **61**, 120–122.

STATISTICS SWEDEN (1990). *Population Statistics*. Stockholm, 1990.

STEINBERG, K.K., THACKER, S.B., SMITH, S.J., STROUP, D.F., ZACK, M.W., FLANDERS, W.D. & BERKELMAN, R.L. (1991). A meta-analysis of the effect of estrogen replacement therapy on the risk of breast cancer. *JAMA*, **265**, 1985–1990.

STJERNSWÄRD, S.K.J. & KOROLTCHOUK, V. (1988). Cancers of the stomach, lung and breast: mortality trends and control strategies. *Wld. Hlth. Statist.*, **Q41**, 107–114. (WHO).

TABAR, L., FAGERBERG, C., GAD, A., BALDETORP, L., HOLMBERG, L., GRÖNTOFT, O., LJUNGQUIST, U., LUNDSTRÖM, B. & MÅNSON, J.C. (1985). Reduction in mortality from breast cancer after mass screening with mammography. *Lancet*, **i**, 829–832.

UK NATIONAL CASE CONTROL STUDY GROUP (1989). Oral contraceptive use and breast cancer risk in young women, *Lancet*, **i**, 973–982.

WHITE, E., DALING, J.R., NORSTED, T.L. & CHU, J. (1987). Rising incidence of breast cancer among young women in Washington state. *JNCI*, **2**, 239–243.

WHITE, E., LEE, C.Y. & KRISTAL, A.R. (1990). Evaluation of the increase in breast cancer incidence in relation to mammography use. *JNCI*, **82**, 1546–1552.